# Infections and mixed infections with the selected species of *Borrelia burgdorferi* sensu lato complex in *Ixodes ricinus* ticks collected in eastern Poland: a significant increase in the course of 5 years

**DOI:** 10.1007/s10493-015-9990-4

**Published:** 2015-11-21

**Authors:** Angelina Wójcik-Fatla, Violetta Zając, Anna Sawczyn, Jacek Sroka, Ewa Cisak, Jacek Dutkiewicz

**Affiliations:** Department of Health Biohazards and Parasitology, Institute of Rural Health, Jaczewskiego 2, 20-090 Lublin, Poland; Department of Parasitology and Invasive Diseases, National Veterinary Research Institute, Pulawy, Poland

**Keywords:** *Ixodes ricinus*, *Borrelia burgdorferi* sensu lato, *Borrelia burgdorferi* sensu stricto, *Borrelia garinii*, *Borrelia afzelii*, Mixed infections, Eastern Poland

## Abstract

In the years 2008–2009 and 2013–2014, 1620 and 1500 questing *Ixodes ricinus* ticks, respectively, were examined on the territory of the Lublin province (eastern Poland). The presence of three pathogenic species causing Lyme disease was investigated: *Borrelia burgdorferi* sensu stricto, *B. afzelii* and *B. garinii*. The proportion of *I. ricinus* ticks infected with *B. burgdorferi* sensu lato showed a highly significant increase between 2008–2009 and 2013–2014, from 6.0 to 15.3 %. A significant increase was noted with regard to all types of infections with individual species: single (4.7–7.8 %), dual (1.2–6.6 %), and triple (0.1–0.9 %). When expressed as the percent of all infections, the frequency of mixed infections increased from 21.4 to 49.2 %. Statistical analysis performed with two methods (by calculating of odds ratios and by Fisher’s exact test) showed that the frequencies of mixed infections in most cases proved to be significantly greater than expected. The strongest associations were found between *B. burgdorferi* s. s. and *B. afzelii*, and between *B. burgdorferi* s. s. and *B. garinii*. They appeared to be highly significant (*P* < 0.0001) when assessed by two methods for 2013–2014, and for the sum of findings for both time periods. The proportions of the individual species detected in the mixed infections in 2008–2009 and 2013–2014 revealed highly significant increases for *B. burgdorferi* s. s. and *B. garinii* (from 33.9 to 71.1 % and from 18.2 to 82.9 %, respectively), and an insignificant decrease for *B. afzelii* (from 51.4 to 41.6 %). The proportions of the species *B. burgdorferi* s. s., *B. afzelii* and *B. garinii* (with combined single and mixed infections) for 2008–2009 and 2013–2014 were: 51.2/44.0 %, 30.6/24.9 % and 18.2/31.1 %, respectively. In conclusion, our results seem to indicate the detrimental trend of the increasing infection rate of *I. ricinus* ticks with *B. burgdorferi* s. l. in eastern Poland, and dramatic enhancement of mixed infections with individual species, which may result in mixed infections of humans and exacerbation of the clinical course of Lyme disease cases on the studied area.

## Introduction

The spirochetes of *Borrelia burgdorferi* sensu lato complex, transmitted mostly by ticks belonging to *Ixodes* genus, cause Lyme borreliosis, a multisystemic disorder which is regarded as the most abundant tick-borne disease of humans worldwide, although it only occurs in the northern hemisphere (Rizzoli et al. 2014). Currently, 19 different species were identified within *B. burgdorferi* s. l. complex, of which at least 9 (*B. burgdorferi* sensu stricto, *B. afzelii*, *B. garinii*, *B. valaisiana*, *B. spielmanii*, *B. bavariensis*, *B. bissettii*, *B. finlandensis*, *B. carolinensis*, and *B. lusitaniae*) are present in Europe (Lommano et al. 2012; Rizzoli et al. 2014). The most important from the viewpoint of human medicine are 3 species: *B. burgdorferi* s. s. mainly associated with Lyme arthritis, *B. garinii* preferentially associated with neuroborreliosis, and *B. afzelii* mostly attributed to skin manifestations (Rauter and Hartung 2005; James et al. 2014; Rizzoli et al. 2014; Martin et al. 2015). Thus, determining of the species spectrum occurring in the tick vectors and/or vertebrate hosts of *B. burgdorferi* s. l. is important for the prognosis and prevention of Lyme borreliosis in particular regions of Europe. Of special relevance is identification of the proportion of polymicrobial infections appearing in one tick or vertebrate host, which increase the severity of disease symptoms when transmitted to humans or animals (Ginsberg 2008; Lommano et al. 2012). Polymicrobial infections may occur as mixed infections involving species of the same genus, such as different species of *B. burgdorferi* s. l. complex, or co-infections involving species of different genera, such as *B. burgdorferi* s. l. and other species causing human tick-borne diseases, such as *Anaplasma phagocytophilum* causing granulocytic anaplasmosis, *Babesia microti* or *Babesia divergens* causing babesiosis, *Bartonella* spp. causing bartonellosis, and *Rickettsia* spp. causing spotted fever (Lommano et al. 2012).

According to many authors, individual species of the *B. burgdorferi* s. l. complex are associated with different vertebrate hosts: *B. afzelii* with rodents, *B. garinii* and *B. valaisiana* with birds, and *B. burgdorferi* s. s. with both birds and rodents (Kurtenbach et al. 2001; James et al. 2014). The resistance and/or sensitivity to the host’s complement is regarded as an important factor in these associations: for example, *B. afzelii* is resistant to the complement of rodents, but could be lysed by the complement of birds (Kurtenbach et al. 2001). Therefore, mixed reactions between the “rodent” and “bird” species are supposed to be less frequent.

In the hitherto published articles there are many reports on the observed mixed infections between the *B. burgdorferi* s. l. species, but not all of them were analyzed statistically and determined as positive (number greater than expected) or negative (number smaller than expected). Ginsberg (2008) reviewed reports published to-date on mixed infections between the *B. burgdorferi* s. l. species, and found 9 significantly positive and 4 significantly negative out of 27 analyzed associations in *Ixodes ricinus* and 3 significantly positive out of 3 analyzed associations in *I. persulcatus*. Most commonly were reported associations between *B. afzelii* and *B. garinii* with 4 significantly positive and 1 significantly negative out of 11 analyzed. According to meta-analysis performed by Rauter and Hartung (2005), the combination of *B. garinii* and *B. valaisiana* occurred in Europe 51 % more often than all other species combinations, but this is not supported by statistical data. In the material of Kurtenbach et al. (2001) from 5 European countries, *B*. *garinii* and *B*. *valaisiana* constituted the majority of multiple infections, whereas the combination of *B*. *garinii* and *B*. *afzelii* occurred significantly less frequently than expected.

The aim of the presented study was to determine the prevalence of 3 species belonging to the *B. burgdorferi* s. l. complex (*B. burgdorferi* s. s., *B. garinii*, and *B. afzelii*) in questing *I. ricinus* ticks collected in the Lublin region (eastern Poland) in 2 time periods, separated by a 5-year interval (2008–2009 and 2013–2014), with special attention being paid to the occurrence of mixed infections. The species were selected for the study because of their afore-mentioned significance as primary agents causing Lyme borreliosis. To assess in the most reliable way possible to determine whether the observed mixed infections between these species occur more or less often than expected, and whether these relations are statistically significant, two statistical methods were applied—the Odds Ratio calculation and Fisher’s exact test.

## Materials and methods

### Collection of ticks

**Fig. 1:**
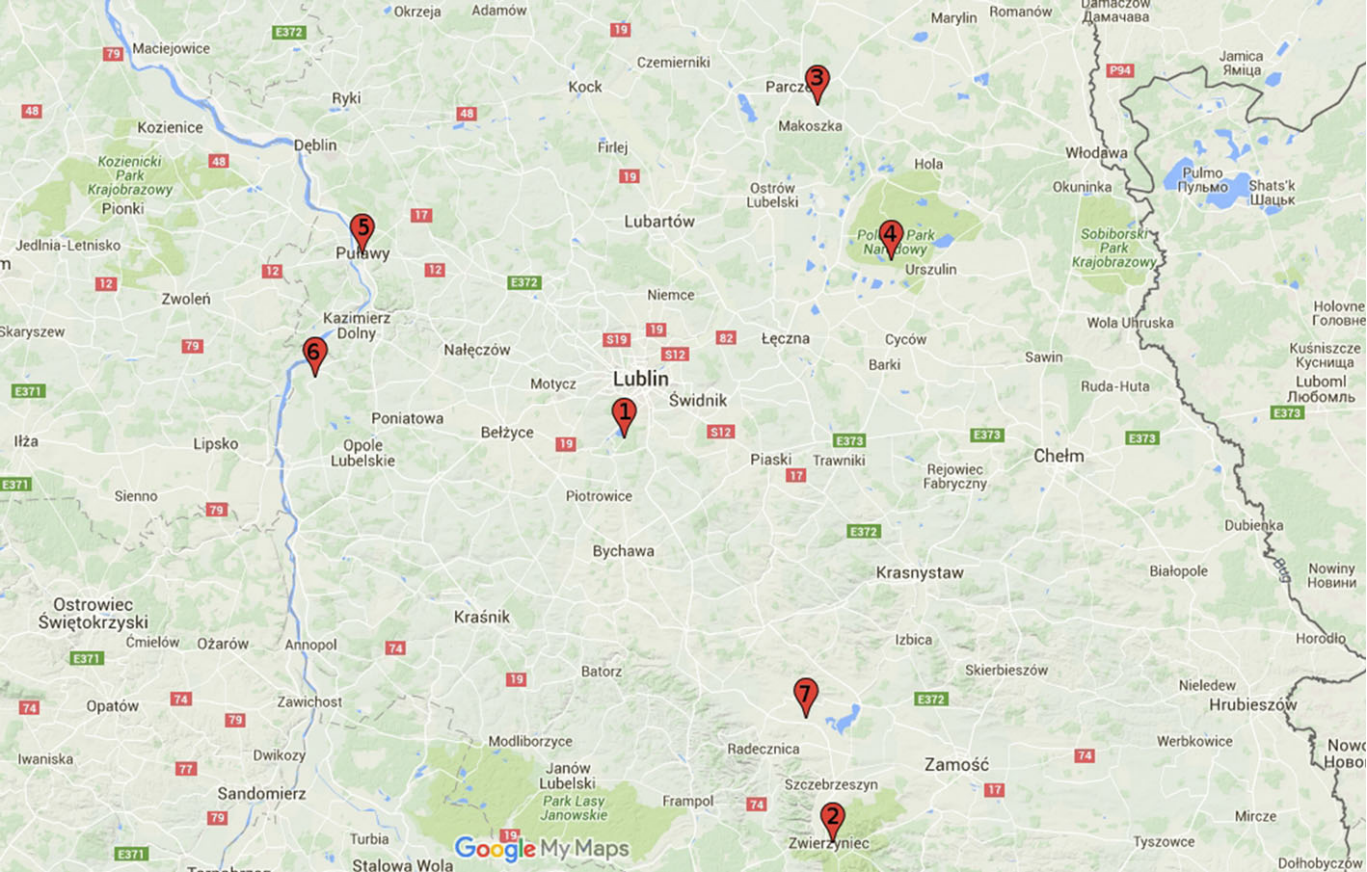
Map presenting localities of ticks collection in the years 2008–2009 and 2013–2014. Numbers of localities correspond to those mentioned in “Materials and methods” and in Table 3: *1* Dąbrowa, *2* Zwierzyniec, *3* Parczew, *4* Łęczna-Włodawa Lake District, *5* Puławy, *6* Wilków, *7* Nielisz

In 2008–2009 (April–September 2008 and 2009), a total of 1620 questing *I. ricinus* ticks (685 nymphs, 471 females and 464 males) were collected on the territory of 5 localities situated in the Lublin province: (1) Dąbrowa (51°10′N 22°32′E), (2) Zwierzyniec (50°37′N 22°59′E), (3) Parczew (51°37′N, 22°57′E), (4) Łęczna-Włodawa Lake District (51°30′N 23°24′E) and (5) Puławy (51°25′N 21°58′E) (Fig. 1).

Collection sites 1 and 3—mixed forests with prevalence of deciduous trees species. Sites 2 and 5-mixed and coniferous forests, with prevalence of conifer trees. Site 4: lake area with prevalence of conifer trees. In 2013–2014 (April–October 2013 and May–September 2014), a total 1500 questing *I. ricinus* ticks (591 nymphs, 467 females and 442 males) were collected from 6 localities: (1) Dąbrowa, (2) Zwierzyniec, (3) Parczew, (4) Łęczna-Włodawa Lake District, (5) Wilków (51°15′N, 21°52′E) and (6) Nielisz (51°47′N 23°02′E) (Fig. 1).

Sites 1, 3, 6, 7—mixed forests with prevalence of decidous trees. Site 2 and 5—mixed and coniferous forests with prevalence of confiers. Site 4: lake area with prevalence of conifers.

Ticks were collected by the flagging of lower vegetation and litter along the paths and edges of deciduous and mixed forests. Collected ticks were placed alive in glass tubes for further investigation.

They were identified according to the monograph by Siuda (1993).

### DNA isolation from ticks

Total DNA was isolated from adult ticks separately, and from nymphs, in pools of 5 specimens by boiling in 0.7 M ammonium hydroxide and stored at −20 °C for further analysis (Rijpkema et al. 1996). Prevalence of infection in nymphs was expressed as the minimum infection rate (MIR) of pools, calculated according to Kahl et al. (1989). The concentration of DNA in the isolates was determined with a NanoDrop ND1000 Spectrophotometer (USA). The determined DNA concentrations ranged from 190 to 480 ng/μl for adult ticks and 20–50 ng/μl for nymphs of *I. ricinus*.

### Detection of *Borrelia burgdorferi* sensu lato by PCR

The isolates were examined for the presence of *B. burgdorferi* s. l. DNA by polymerase chain reaction (PCR) with primer set FLA1 (5′-AGAGCAACTTACAGACGAAATTAAT-3′) and FLA2 (5′-CAAGTCTATTTTGGAAAGCACCTAA-3′) specific for DNA *fla* gene sequence (Stańczak et al. 2000; Wodecka and Sawczuk 2004) according to the method described previously (Wójcik-Fatla et al. 2009), with some modifications. Each PCR reaction was carried out in a 20 μl reaction volume which contained the following mix of reagents: 0.5 U *Taq* DNA polymerase (Qiagen, USA), 1 × PCR buffer containing 15 mM MgCl_2_, 0.5 μl 2 mM dNTP (final concentration 0.05 mM) (Thermo Scientific, Lithuania), 0.8 μl 10 μM each of primer (Eurogentec, Seraing, Belgium), 2 μl of matrix DNA and nuclease-free water (Applied Biosystems, USA). The amplification was carried out in C1000 Thermal Cycler (BioRad, USA) under the following conditions: preincubation at 95 °C for 3 min, 35 cycles, each of 30 s at 94 °C (denaturation), 45 s at 54 °C (primers annealing), and 45 s at 72 °C (elongation). Products of amplification were identified in 2 % agarose gel (Prona, Basica LE), after electrophoresis in standard conditions and staining with ethidium bromide solution (2 μg/ml). Nuclease-free water was used as a negative control. Positive control was obtained by culture of *Borrelia* spirochetes isolated from *I. ricinus* ticks collected from vegetation according to methods described previously (Cisak et al. 2006).

### Detection of *Borrelia burgdorferi* s. l. species by nested-PCR

Confirmatory re-amplification by nested-PCR was performed with the method described previously (Stańczak et al. 2000; Wodecka and Sawczuk 2004) with some modification. As mentioned earlier, the following 3 species known as primary agents causing Lyme borreliosis were selected for the study: *B. burgdorferi* s. s., *B. afzelii* and *B. garinii*. Nested-PCR reaction was carried out in the volume of 20 μl which contained: 0.5 U *Taq* DNA polymerase (Qiagen, USA), 1 × PCR buffer containing 15 mM MgCl_2_, 0.5 μl 2 mM dNTP (Fermentas, Lithuania), 1.2 μl 10 μM each of primer (Eurogentec, Seraing, Belgium), 2 μl of the first amplification product and nuclease-free water (Applied Biosystems, USA). The following primer sets were used for the detection of species: BB1 (5′-AATCTTTTCTCTGGTGAG-3′) and BB2 (5′-GAGCTCCTTCCTGTTGAA-3′) for *B. burgdorferi* s. s., BA1 (5′-ATGTTGCAAATCTTTTTG-3′) and BA2 (5′-TAGCAGGTGTTGGTTGCT-3′) for *B. afzelii*, BG1 (5′-AATCTATTCTCTGGCGAA-3′) and BG3 (5′-GGAGAATTAACTCCACCC-3′) for *B. garinii*. Reamplification was carried out in a C1000 Thermal Cycler (BioRad, USA) under the following conditions: preincubation at 94 °C for 60 s, 30 cycles, each of 30 s at 94 °C, 30 s at 58 °C (for *B. burgdorferi* s. s. and *B. afzelii*), or 30 s at 60 °C (for *B. garinii*), and 60 s at 72 °C. The sizes of amplified DNA fragments were: 76 bp for *B. burgdorferi* s. s., 103 bp for *B. afzelii* and 125 bp for *B. garinii*. Amplification products were identified in 3.5 % agarose gel, after electrophoresis in standard conditions and staining with ethidium bromide solution (2 μg/ml).

### Statistical analysis

The results concerning prevalence of individual species in examined ticks were analyzed by χ^2^ test and Student’s *t* test, using the STATISTICA v. 6.0 package (Statsoft, Tulsa, OK, USA). The significance of mixed infections by a comparison to expected values was assessed with two methods: Odds Ratio calculation using MedCalc® software (MedCalc 2015) and by Fisher’s exact test using GraphPad software (GraphPad 2015). The value *P* < 0.05 was considered as significant.

## Results

### Prevalence of total infections with *Borrelia burgdorferi* s. l. and mixed infections with individual species in 2008–2009 and 2013–2014

As seen in Table 1, the proportion of *I. ricinus* ticks infected with *B. burgdorferi* s. l. and individual species (*B. burgdorferi* s. s., *B. afzelii* and *B. garinii*) showed a highly significant increase in 2013–2014 compared to 2008–2009, from a total of 6.0 % ticks infected with *B. burgdorferi* s. l. in 2008–2009 to 15.3 % in 2013–2014 (*P* < 0.0001). A significant increase was also noted with regard to all types of infections for all 3 genospecies: single (4.7–7.8 %; *P* = 0.0003), dual (1.2–6.6 %; *P* < 0.0001), and triple (0.1–0.9 %; *P* < 0.0013). Detailed analysis of individual associations with the single infections revealed a highly significant increase in the prevalence of infections with *B. afzelii* (*P* < 0.0001), while the differences between the prevalence of infections with *B. burgdorferi* s. s. and *B. garinii* were not significant. By contrast, at the dual infections, a highly significant increase was found with the mixed infections of *B. burgdorferi* s. s. and *B. garinii* (0.1–5.1 % of the total ticks examined), whereas the increase of the remaining 2 associations was insignificant (Table 1).

**Table 1:** Differences between prevalence of infections and mixed infections with *Borrelia burgdorferi* sensu lato species (number of infected specimens, percent) in the *Ixodes ricinus* ticks collected in eastern Poland in two time periods: in the years 2008–2009 and in the years 2013–2014

Species time period	Single infections				Dual infections				Triple infections	Total mixed infections with *B. b.* s. l. species	Total infections and mixed infections with *B. b.* s. l. species
	*B. b.* s. s.	*B. afzelii*	*B. garinii*	Total single infections	*B. b.* s. s. + *B. afzelii*	*B. b.* s. s. + *B. garinii*	*B. afzelii* + *B. garinii*	Total dual infections	*B. b.* s. s. + *B. afzelii* + *B. garinii*		
*Adults*											
Years 2008–2009 N = 785	33 (4.2 %)	15 (1.9 %)	14 (1.8 %)	62 (7.9 %)	14 (1.7 %)	2 (0.3 %)	0 (0)	16 (2.0 %)	2 (0.3 %)	18 (2.3 %)	80 (10.2 %)
Years 2013–2014 N = 909	36 (4.0 %)	28 (3.1 %)	15 (1.6 %)	79 (8.7 %)	17 (1.9 %)	60 (6.6 %)	0 (0)	77 (8.5 %)	10 (1.1 %)	87 (9.6 %)	166 (18.3 %)
	*P* = 0.86 NS	*P* = 0.12 NS	*P* = 0.75 NS	*P* = 0.51 NS	0.76 NS	*P* < 0.0001 ++	*P* = 1.0 NS	*P* < 0.0001 ++	*P* = 0.054 NS	*P* < 0.0001 ++	*P* < 0.0001 ++
*Nymphs*											
Years 2008–2009 N = 835	8 (0.9 %)	3 (0.4 %)	4 (0.5 %)	15 (1.8 %)	3 (0.4 %)	0 (0)	0 (0)	3 (0.4 %)	0 (0)	3 (0.4 %)	18 (2.2 %)
Years 2013–2014 N = 591	10 (1.7 %)	24 (4.0 %)	4 (0.7 %)	38 (6.4 %)	4 (0.7 %)	16 (2.7 %)	2 (0.3 %)	22 (3.7 %)	4 (0.7 %)	26 (4.4 %)	64 (10.8 %)
	*P* = 0.18 NS	*P* < 0.0001 ++	*P* = 0.63 NS	*P* < 0.0001 ++	0.44 NS	*P* < 0.0001 ++	*P* = 0.11 NS	*P* < 0.0001 ++	*P* = 0.016 +	*P* < 0.0001 ++	*P* < 0.0001 ++
*Total ticks*											
Years 2008–2009 N = 1620	41 (2.5 %)	18 (1.1 %)	18 (1.1 %)	77 (4.7 %)	17 (1.1 %)	2 (0.1 %)	0 (0)	19 (1.2 %)	2 (0.1 %)	21 (1.3 %)	98 (6.0 %)
Years 2013–2014 N = 1500	46 (3.1 %)	52 (3.5 %)	19 (1.3 %)	117 (7.8 %)	21 (1.4 %)	76 (5.1 %)	2 (0.1 %)	99 (6.6 %)	14 (0.9 %)	113 (7.5 %)	230 (15.3 %)
	*P* = 0.31 NS	*P* < 0.0001 ++	*P* = 0.61 NS	*P* = 0.0003 +	0.45 NS	*P* < 0.0001 ++	*P* = 0.20 NS	*P* < 0.0001 ++	*P* = 0.0013 +	*P* < 0.0001 ++	*P* < 0.0001 ++

*B. b.* s. s. *Borrelia burgdorferi* sensu stricto; *B. b.* s. l. *B. burgdorferi* sensu lato; *N* number of examined specimens

*NS* difference between time periods not significant; +, difference between time periods significant (*P* < 0.05); ++, difference between time periods highly significant (*P* < 0.0001)

When expressed as the percent of all infections, the frequency of mixed infections showed a highly significant increase from 21.4 % in 2008–2009 to 49.2 % in 2013–2014 (*P* < 0.0001). The proportions of the individual species detected in the mixed infections in 2008–2009 and 2013–2014 revealed a highly significant increases (*P* < 0.0001) for *B. burgdorferi* s. s. and *B. garinii* (33.9–71.1 % and from 18.2 to 82.9 %, respectively), while an insignificant decrease (51.4–41.6 %) was noted only for *B. afzelii*.

### Differences between the prevalence of total infections with *Borrelia burgdorferi* s. l. in nymphs and adults

The infection rates in adult ticks were significantly greater than in nymphs for both time periods (Table 1). In 2008–2009, the prevalence of mixed infections in adults versus nymphs was 2.3 versus 0.4 % (*P* = 0.0008) while the prevalence of total infections was 10.2 versus 2.2 % (*P* < 0.0001). In 2013–2014, the corresponding data were 9.6 versus 4.4 % (*P* = 0.0002) for mixed infections and 18.3 versus 10.8 % (*P* < 0.0001) for total infections.

Similarly as in total ticks, also in nymphs, highly significant increases in the prevalence of infection during 2008–2009–2013–2014 were noted for single infections (1.8–6.4 %; *P* < 0.0001), mixed infections (0.4–4.4 %, *P* < 0.0001), and total infections (2.2–10.8 %; *P* < 0.0001). In adult ticks, the increase was not significant for single infections (7.9–8.7 %; *P* = 0.51), but appeared to be significant for mixed infections (2.3–9.6 %; *P* < 0.0001) and total infections (10.2–18.3 %; *P* < 0.0001). When expressed as a percent of total infections, the proportion of mixed infections increased between time periods from 16.7 to 40.6 % in nymphs and from 22.5 to 52.4 % in adults.

### Significance of mixed infections with individual species of *Borrelia burgdorferi* s. l. in *Ixodes ricinus*

As seen in Table 2, the frequencies of mixed infections for total ticks, assessed by two statistical methods, in most cases proved to be significantly greater than expected, which means that the infection with one species significantly increased the chance for infection with the second species. The strongest association was found between *B. burgdorferi* s. s. and *B. afzelii*, which appeared to be highly significant (*P* < 0.0001) when assessed by two methods for 2008–2009 and 2013–2014, and for the sum of findings for both time periods. The relationships for other two associations were also significant, with only one exception for the association between *B. afzelii* and *B. garinii*, which did not attain the significance level when assessed by Fisher’s exact test for 2008–2009. The association between *B. burgdorferi* s. s. and *B. garinii* was highly significant (*P* < 0.0001) when assessed by two methods for 2013–2014 and for the total count, with the extremely high values of OR −77.3 and 188.2, respectively. The mixed infections proved to be also significantly greater than expected when analysed separately for nymphs and adult ticks, except for the associations between *B. burgdorferi* s. s. and *B. garinii* and between *B. afzelii* and *B. garinii* in 2008–2009. Neither of them attained the significance levels for nymphs, while the association between *B. afzelii* and *B. garinii* was not significant for adults (Table 2).

**Table 2:** Mixed infections with *Borrelia burgdorferi* s.l. species in *Ixodes ricinus* ticks: assessment of significance with two statistical methods, by computing of odds ratio and by Fisher’s exact test

Assessed mixed infection time period	*B. burgdorferi* s. s. versus *B. afzelii*		*B. burgdorferi* s. s. versus *B. garinii*		*B. afzelii* versus *B. garinii*	
*Adults*						
Years 2008–2009	C 16 (2.0 %)		C 4 (0.5 %)		C 2 (0.3 %)	
	OR 21.9	95 % CI 10.0–47.9	OR 4.3	95 % CI 1.4–13.5	OR 3.2	95 % CI 0.7–14.5
N = 785	P_OR_ < 0.0001	P_Fisher_ < 0.0001	P_OR_ = 0.013	P_Fisher_ = 0.024	P_OR_ = 0.14	P_Fisher_ = 0.16
Years 2013–2014	C 27 (3.0 %)		C 70 (7.7 %)		C 10 (1.1 %)	
	OR 7.6	95 % CI 4.3–13.5	OR 67.9	95 % CI 36.4–126.6	OR 2.3	95 % CI 1.1–4.8
N = 909	P_OR_ < 0.0001	P_Fisher_ < 0.0001	P_OR_ < 0.0001	P_Fisher_ < 0.0001	P_OR_ = 0.024	P_Fisher_ = 0.029
Total	C 43 (2.5 %)		C 74 (4.4 %)		C 12 (0.7 %)	
	OR 11.3	95 % CI 7.1–17.8	OR 38.0	95 % CI 23.7–61.2	OR 8.8	95 % CI 4.3–18.0
N = 1694	P_OR_ < 0.0001	P_Fisher_ < 0.0001	P_OR_ < 0.0001	P_Fisher_ < 0.0001	P_OR_ < 0.0001	P_Fisher_ = 0.0046
*Nymphs*						
Years 2008–2009	C 3 (0.4 %)		C 0 (0.0)		C 0 (0.0)	
	OR 102.6	95 % CI 17.9–587.8	OR 10.8	95 % CI 0.5–216.0	OR 26.3	95 % CI 1.2–586.6
N = 835	P_OR_ < 0.0001	P_Fisher_ < 0.0001	P_OR_ = 0.12	P_Fisher_ = 1.0	P_OR_ = 0.039	P_Fisher_ = 1.0
Years 2013–2014	C 8 (1.4 %)		C 20 (3.4 %)		C 6 (1.0 %)	
	OR 28.3	95 % CI 9.1–87.4	OR 131.2	95 % CI 45.7–376.9	OR 5.8	95 % CI 2.1–15.5
N = 591	P_OR_ < 0.0001	P_Fisher_ < 0.0003	P_OR_ < 0.0001	P_Fisher_ < 0.0001	P_OR_ = 0.0005	P_Fisher_ = 0.0023
Total	C 11 (0.8 %)		C 20 (1.4 %)		C 6 (0.4 %)	
	OR 11.6	95 % CI 5.4–26.4	OR 77.8	95 % CI 33.9–178.3	OR 10.0	95 % CI 3.8–26.1
N = 1426	P_OR_ < 0.0001	P_Fisher_ < 0.0001	P_OR_ < 0.0001	P_Fisher_ < 0.0001	P_OR_ < 0.0001	P_Fisher_ < 0.0001
*Total ticks*						
Years 2008–2009	C 19 (1.2 %)		C 4 (0.2 %)		C 2 (0.1 %)	
	OR 37.8	95 % CI 18.5–77.8	OR 5.9	95 % CI 1.9–18.0	OR 5.0	95 % CI 1.1–22.2
N = 1620	P_OR_ < 0.0001	P_Fisher_ < 0.0001	P_OR_ = 0.002	P_Fisher_ = 0.0085	P_OR_ = 0.036	P_Fisher_ = 0.074
Years 2013–2014	C 35 (2.3 %)		C 90 (6.0%)		C 16 (1.0%)	
	OR 6.8	95% CI 4.3–9.9	OR 77.3	95% CI 45.4–131.3	OR 3.0	95% CI 1.7–5.4
N = 1500	P_OR_ < 0.0001	P_Fisher_ < 0.0001	P_OR_ < 0.0001	P_Fisher_ < 0.0001	P_OR_ = 0.0002	P_Fisher_ = 0.0005
Total	C 54 (1.7%)		C 94 (3.0%)		C 18 (0.6%)	
	OR 17.7	95 % CI 11.9–26.6	OR 188.2	95 % CI 110.4–320.7	OR 6.3	95 % CI 3.7–11.0
N = 3120	P_OR_ < 0.0001	P_Fisher_ < 0.0001	P_OR_ < 0.0001	P_Fisher_ < 0.0001	P_OR_ < 0.0001	P_Fisher_ < 0.0001

*N* Number of examined ticks; *C* number of mixed infections and percent related to the total tick population examined; *OR* odds ratio, calculated according to formula given by MedCalc 2015; *95* *% CI* 95 % confidence intervals for OR; *P*_*OR*_ probability of mixed infection with relation to expected value assessed by odds ratio; *P*_*Fisher*_ Probability of mixed infection with relation to expected value assessed by Fisher’s exact test

### Species composition of *Borrelia burgdorferi* s. l. strains detected in *Ixodes ricinus* in 2008–2009 and 2013–2014

In 2008–2009, the most prevalent species was *B. burgdorferi* s. s. which accounted for the majority of *B. burgdorferi* s. l. strains (51.2 %) while the frequencies of *B. afzelii* and *B. garinii* were 30.6 and 18.2 %, respectively. In 2013–2014, a considerable increase in *B. garinii* proportion to 31.1 % was noted, whereas the proportions of *B. burgdorferi* s. s. and *B. afzelii* slightly decreased to 44.0 and 24.9 %, respectively.

### The locality-dependent variability in infection rates

By χ^2^ test, variability between individual localities appeared significant in the case of all species and time periods, except for *B. afzelii* in 2008–2009 (Table 3). For 2013–2014, variability appeared to be highly significant (*P* < 0.00001) for all *B. burgdorferi* s. l. species. The increases in the infection rate and mixed infections between 2008–2009 and 2013–2014 were not uniform in all the localities, and were highly significant only in Dąbrowa (4.5–24.3 %; *P* < 0.00001) and in Puławy region on the Vistula river (4.9 % in Puławy–25.0 % in Wilków; *P* < 0.00001). The differences observed in other localities were not significant (*P* > 0.05).

**Table 3:** The infection of *Ixodes ricinus* ticks with individual species of *Borrelia burgdorferi* sensu lato in particular localities (number of infected specimens, percent)

Species time period	Locality	*B. burgdorferi* s. s.*	B. afzelii*	B. garinii*	Total *B. burgdorferi* s.l.**
2008–2009	1. Dąbrowa N = 556	14 (2.5 %)	13 (2.3 %)	2 (0.4 %)	25 (4.5 %)
	2. Zwierzyniec N = 257	7 (2.7 %)	4 (1.6 %)	5 (1.9 %)	15 (5.8 %)
	3. Parczew N = 245	18 (7.3 %)	9 (3.7 %)	11 (4.5 %)	31 (12.7 %)
	4. Łęczna-Włodawa Lake District N = 153	5 (3.3 %)	2 (1.3 %)	2 (1.3 %)	7 (4.6 %)
	5. Puławy N = 409	17 (4.2 %)	9 (2.2 %)	2 (0.5 %)	20 (4.9 %)
	Total N = 1620	62 (3.8 %)	39 (2.4 %)	22 (1.4 %)	98 (6.0 %)
	Assessment of the locality-dependent variability (χ^2^ test)	χ^2^ = 11.5833 *P* = 0.021 +	χ^2^ = 3.2626 *P* = 0.51 NS	χ^2^ = 24.2520 *P* = 0.00007 ++	χ^2^ = 21.5437 *P* = 0.00025 ++
2013–2014	1. Dąbrowa N = 404	71 (17.6 %)	48 (11.9 %)	52 (12.9 %)	98 (24.3 %)
	2. Zwierzyniec N = 226	7 (3.1 %)	5 (2.2 %)	2 (0.9 %)	11 (4.9 %)
	3. Parczew N = 235	23 (9.8 %)	11 (4.7 %)	15 (6.4 %)	31 (13.2 %)
	4. Łęczna-Włodawa Lake District N = 218	3 (1.4 %)	1 (0.5 %)	0 (0)	4 (1.8 %)
	6. Wilków N = 316	48 (15.2 %)	21 (6.6 %)	37 (11.7 %)	79 (25.0 %)
	7. Nielisz N = 101	5 (5.0 %)	3 (3.0 %)	4 (4.0 %)	7 (6.9 %)
	Total N = 1500	157 (10.5 %)	89 (5.9 %)	111 (7.4 %)	230 (15.3 %)
	Assessment of the locality-dependent variability (χ^2^ test)	χ^2^ = 58.0251 *P* < 0.00001 ++	χ^2^ = 43.1786 *P* < 0.00001 ++	χ^2^ = 55.2429 *P* < 0.00001 ++	χ^2^ = 88.0497 *P* < 0.00001 ++

*N* number of examined ticks

* Multiple infections are presented separately for each species

** Multiple infections are presented jointly

*NS* variability not significant; +, variability significant (*P* < 0.05); ++, variability highly significant (*P* < 0.001)

## Discussion

Within the period of 5 years under study, we observed on the territory of eastern Poland a highly significant, circa. 2.5-times, increase both in the prevalence of *B. burgdorferi* s. l. infection of *I. ricinus* ticks, and in the proportion of mixed infections which attained an exceptionally high level of nearly a half (49.1 %) of all infected ticks. This finding corresponds well with the statement of Coipan et al. (2013), that over the last decades the incidence of Lyme borreliosis has increased significantly in Europe, and the official health statistics in Poland show an increase in Lyme borreliosis cases from 8255 in 2008 to 13,875 in 2014 (National Institute of Public Health 2015). According to Schwarz et al. (2012), *Borrelia* prevalence has increased during the last decades in different regions of Europe, such as Denmark and Germany. Similar to the present work, these authors noted a significant growth of mixed infections between 2001 and 2007 in *I. ricinus* ticks collected in the Siebengebirge in Germany, and report on a similar increase in Ireland and Denmark (Schwarz et al. 2012). Kampen et al. (2004), in another study carried out in the Siebengebirge in 2001, found that the infection prevalence of *B. burgdorferi* s. l. in questing nymphs and adults of *I. ricinus* was 2.5-fold higher than in the earlier study performed over 10 years earlier, in 1987–1988. On the other hand, Tappe et al. (2014) found between 2005 and 2010 in *I. ricinus* ticks collected in Hanover, Germany, a significant increase in *B. burgdorferi* s.l. infection rate in larvae, but not in adults and nymphs. Also, Rauter and Hartung (2005), in a meta-analysis, did not observe the prevalence of *B. burgdorferi*. s. l. in *I. ricinus* ticks a tendency to increase over time in Europe, and report that no difference was found between collection periods 1986–1993 versus 1994–2002. Thus, most probably the increase observed by us in the infection rate and mixed infections has a focal character and does not occur evenly over the whole area of Europe. The focal character of the tick infection with *B. burgdorferi* s.l. could be observed not only on the levels of countries or regions, but also within regions. Thus, the rapid growth of the infection observed in the Lublin region over the period of 5 years was due to the great increase in suburban Dąbrowa area and northwestern area of the region neighbouring the Vistula river and the localities of Puławy and Wilków. The reason for this is not fully known, it may be supposed that the growing recreational activity in Dąbrowa has resulted in an increase in the number of people and domestic animals serving as potential hosts for ticks. Wilków is situated on the Vistula river in a region exposed to flooding, which might have an impact on the dissemination of infected ticks.

The mean infection rate of *I. ricinus* ticks with *B. burgdorferi* s. l. compiled by Rauter and Hartung (2005) for studies carried our between 1984 and 2003 in Europe, was 13.7 %, which was higher compared to our result obtained in 2008–2009 (6.0 %), and slightly lower compared to that achieved in 2013–2014 (15.3 %). The same applies to infection rates ranging from 11.1 to 13.9 % reported by Kiewra et al. (2014) and Sytykiewicz et al. (2012) from western and central Poland, by Hildebrandt et al. (2003) and Kampen et al. (2004) from Germany, and by Coipan et al. (2013) from the Netherlands (11.8 %). Our results for both time periods are higher compared to those reported by James et al. (2014) from Scotland (5.6 %), but lower compared to those ranging from 16.7 to 48.8 % reported by Skotarczak et al. (2003) from northwestern Poland, Tappe et al. (2014) and Hildebrandt et al. (2010) from Germany, Lommano et al. (2012) from Switzerland, Kurtenbach et al. (2001) from 5 countries including Slovakia, Latvia, Germany, Portugal, the United Kingdom (31.1 %), and by Tomanović et al. (2010) in Serbia (48.8 %). In most of the studies, similar to the present work, adult ticks and nymphs were examined, and only in 2 studies were larvae also investigated (Tappe et al. 2014; James et al. 2014).

In the examined *I. ricinus* ticks, we determined the presence of 3 genospecies of *B. burgdorferi* s. l. which are the most significant agents of Lyme borreliosis. Among them, *B. burgdorferi* s. s. was the most common in both time periods, before *B. afzelii* in 2008–2009 and *B. garinii* in 2013–2014. The genospecies composition observed by us differed from those reported from other parts of Poland (Kiewra et al. 2014), and from other European countries (Kurtenbach et al. 2001; Rauter and Hartung 2005; Strube et al. 2011; Lommano et al. 2012; Coipan et al. 2013; Tappe et al. 2014; James et al. 2014), where *B. afzelii* was reported as the most common genospecies, while *B. burgdorferi* s. s. occurred with much less frequency. Some authors reported *B. garinii* as the most common species (Hildebrandt et al. 2003, 2010), or *B. valaisiana* (Kampen et al. 2004), which were not determined in the ticks we examined. Only Tomanović et al. (2010) reported *B. burgdorferi* s. s. as the most common genospecies of *B. burgdorferi* s. l. in Serbia.

The remarkable increase in mixed infections with different genospecies of *B. burgdorferi* s. l. was noted in the ticks from eastern Poland examined by us: from 21.4 % of all infections in 2008–2009 to 49.2 % in 2013–2014. The frequencies of mixed infections reported hitherto from various European countries are distinctly lower, and in most cases did not exceed even the value from 2008 to 2009, ranging from 0 to 14.3 % (Kurtenbach et al. 2001; Hildebrandt et al. 2003; Kampen et al. 2004; Rauter and Hartung 2005; Tomanović et al. 2010; Hildebrandt et al. 2010; Lommano et al. 2012; Coipan et al. 2013; James et al. 2014; Tappe et al. 2014; Kiewra et al. 2014). Rauter and Hartung (2005) expressed a view that mixed infections of ticks with *B. burgdorferi* s. l. species are rather rare. However, Ginsberg (2008) in another review article quoted higher ranges equal to 0–24.2 % for *I. ricinus* and 5.4–32.9 % for *I. persulcatus*. The high values of mixed infections in *I. ricinus*, ranging from 30.0 to 35.0 % of the total infections with *B. burgdorferi* s. l. noted Strube et al. (2011) and Schwarz et al. (2012) in Germany. Nevertheless, all these values are much lower compared to the value of mixed infections from the years 2013–2014 stated by us, approximating nearly a half of the total infections. This value, which is to the best of our knowledge is the highest value of mixed infections between the *B. burgdorferi* s. l. species ever reported, is largely due to the exceptionally high frequency of the mixed infections between *B. burgdorferi* s. s. and *B. garinii*, equal to 67.3 % of all mixed infections.

In the present study, the frequency of mixed infections between the *B. burgdorferi* s. l. species was very high, particularly in 2013–2014, and significantly higher compared to expected values. The significance level of these relationships was extremely high, mostly for the associations between *B. burgdorferi* s. s. and *B. garinii*, and between *B. burgdorferi* s. s. and *B. afzelii*. Thus, the value of Odds Ratio (OR) for the association between *B. burgdorferi* s. s. and *B. garinii* in 2013–2014 equaled 77.3, what means that the chance of infection with *B. garinii* in the ticks already infected with *B. burgdorferi* s. s. is over 77 times greater than in ticks not infected with *B. burgdorferi* s. s. The level of the associations between the “rodent genospecies” *B. afzelii* and “bird genospecies” *B. garinii*, was relatively lower, although still very significant with an “OR” value equal to 3.0 and “P” value equal to 0.0002 in 2013–2014.

The prominent association between *B. burgdorferi* s. s. and *B. garinii* found in our material has not been reported in earlier publications, with the exception of articles by Hildebrandt et al. (2003, 2010), who found in *I. ricinus* ticks collected in Thuringia (Middle Germany) dual infections with these 2 species, and the study by Alekseev et al. (2003) who found highly significant mixed infections between *B. burgdorferi* s. s. and *B. garinii* in 32.9 % of *I. persulcatus* ticks infected with *B. burgdorferi* s. l. in Russia. These authors also found highly significant positive associations between *B. burgdorferi* s. s and *B. afzelii*, as well as between *B. afzelii* and *B. garinii,* which is also in accordance with the results of our study.

The very high incidence of mixed infections between *B. burgdorferi* s. s. and *B. garinii* stated in *I. ricinus* ticks in our study creates a potential hazard for humans living in eastern Poland and exposed to tick bites. The parallel inoculation of borreliae belonging to both genospecies by infected tick is associated with the risk of developing severe Lyme borreliosis, in which arthritis caused by *B. burgdorferi* s. s. is combined with neuroborreliosis caused by *B. garinii*. Of importance in this context is the study by Hovius et al. (2007) who proved that simultaneous experimental infection with *B. garinii* and *B. burgdorferi* s. s. in mice resulted in more severe Lyme borreliosis. These authors suggested that competition between these 2 *Borrelia* species within the reservoir host could have led to preferential maintenance, and a rising prevalence, of *B. burgdorferi* s. s. in European *I. ricinus* populations. Our findings seem to support this presumption.

In conclusion, our study demonstrates a rapid growth, within the period of 5 years, in the prevalence of infection with *B. burgdorferi* s. l and in the frequency of mixed infections with individual genospecies, in *I. ricinus* ticks occurring on the territory of eastern Poland. Although the prevalence of tick infection at the end of study period (15.3 %) approximates the European average, and by itself is not alarming, the potential hazard is associated with the high rate of its growth (2.5-fold) and, most of all, with the extraordinary and until now unreported increase in the proportion of mixed infections, especially those between *B. burgdorferi* s. s. and *B. garinii*, creating a risk of severe cases of Lyme borreliosis with arthritis and affecting the neural system.
